# Nodular Lymphocyte Predominant Hodgkin Lymphoma of the Thyroid

**DOI:** 10.1155/2016/8756723

**Published:** 2016-12-01

**Authors:** Carlos Tavares Bello, João Cassis, Helder Simões, João Sequeira Duarte

**Affiliations:** ^1^Endocrinology Department, Hospital de Egas Moniz, Lisboa, Portugal; ^2^Pathology Department, Hospital de Egas Moniz, Lisboa, Portugal; ^3^Endocrinology Department, Instituto Português de Oncologia Francisco Gentil, Lisboa, Portugal

## Abstract

Thyroid lymphomas are rare clinical entities that may result from either the primary intrathyroid* de novo* or secondary thyroid gland involvement of a lymphoma. Among these, the Hodgkin's subtype is quite uncommon, accounting for 0.6–5% of all thyroid malignancies. The authors report on a 76-year-old female presenting with a thyroid nodule that, upon surgical excision, was found to be a nodular lymphocyte predominant Hodgkin lymphoma of the thyroid. So far, thyroid involvement by this variant has never been reported. Upon reporting on this clinical case, the authors emphasize the difficulties usually found in establishing the diagnosis and in defining the best management strategy. A thorough review of the available literature is done.

## 1. Introduction

Hodgkin's lymphoma (HL) is a malignant lymphoproliferative disease encompassing a wide spectrum of lymphoid neoplasms. It accounts for 8.2% of all lymphomas [1] and 0.5% of all cancers diagnosed in the developed world annually [2]. In Europe, the incidence is approximately 2.4 per 100.000 per year [3]. It has a bimodal age distribution with the highest peak affecting young adults (around 20 years of age) and a second peak in the elderly (older adults, approximately 65 years). Males are more frequently affected than female patients [1]. Risk factors include the socioeconomic status (high for nodular sclerosis and low for mixed cellularity and lymphocyte depleted subtypes),* Epstein-Barr* virus infection, immunosuppression (iatrogenic or HIV, 5–25-fold higher risk), genetic background (HLA-DRB1 and HLA-DQB1 genotypes), and autoimmunity (Rheumatoid Arthritis, OR 2.7; Systemic Lupus Erythematosus, OR 5.8; Sarcoidosis, OR 14) [4]. HL arises from germinal center or postgerminal center B cells and has a unique cellular composition consisting of a variable amount of neoplastic cells in an inflammatory background. Reed-Sternberg cells and their variants (Hodgkin's, mummified, lacunar, lymphocytic, and histiocytic cells) are diagnostic. The phenotype of the inflammatory cell background infiltrate, along with the neoplastic cell morphological and immunophenotypical features, subdivides HL in 2 subgroups: classical and nodular lymphocyte predominant HL. Classical HL is further subdivided in 4 subtypes: nodular sclerosis (70% of all HL), mixed cellularity (20%), lymphocyte-rich (5%), and lymphocyte-depleted (<1%) HL. Nodular lymphocyte predominant HL (NLPHL) accounts for less than 10% of all HL cases and is considered a distinct clinical entity [4].

HL usually presents with painless lymphadenopathy (70%), mostly cervical in location (60–80%). B symptoms (fever, chills, and weight loss) are present in 20% of patients, may alert systemic involvement, and constitute a poor prognostic marker. Extranodal disease, as opposed to non-Hodgkin lymphomas (present in 33% of cases), is less frequent with HL (5–10%) [3].

Primary Malignant Lymphoma of the Thyroid is uncommon, accounting for less than 5% of all thyroid malignancies and up to 7% of all extranodal lymphomas [5–8]. It tends to be of B cell origin, with the majority being of non-Hodgkin's subtype (MALT and diffuse large B cell subtype). It predominantly affects elderly female patients in their 7th decade of life. Its etiology remains largely unknown but lymphocytic thyroiditis, by allowing lymphoid cell infiltration of the thyroid bed (a lymphoid cell deprived organ), may well be involved. It is often difficult to establish the primary source of HL in patients with both nodal and extranodal involvement; however, primary extranodal forms are extremely rare [9].

The case of a patient bearing a nodular lymphocyte predominant Hodgkin's lymphoma affecting the thyroid gland is reported.

## 2. Case Report

**Figure 1:**
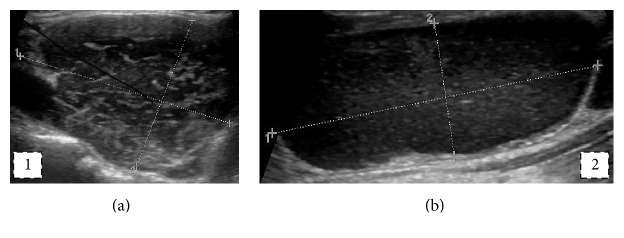
(a) Thyroid nodule, large (29 × 44 × 31 mm transverse, longitudinal, and anteroposterior diameters, resp.) hypoechogenic heterogeneous thyroid nodule with regular borders; (b) lymph node, large (48 × 20 mm), hypoechoic, partly cystic lymph node.

**Figure 2:**
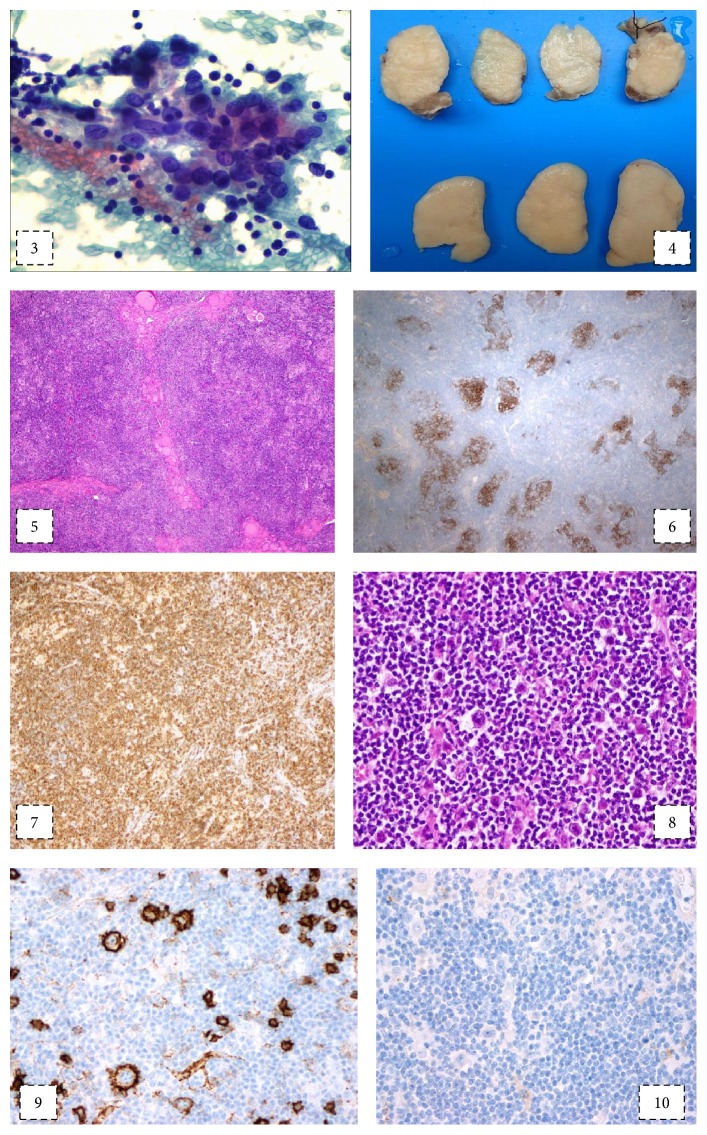
3: FNA of the thyroid with follicular cells with suspicious nuclei (anisokaryosis, longitudinal grooves) on the background of a lymphocytic thyroiditis (Papanicolaou stain, 400x); 4: macroscopy features of the surgical specimens: left hemithyroidectomy (on top) and left cervical lymph node (on the bottom); 5: thyroid parenchyma with a diffuse infiltration of a lymphoid neoplasm (HE, 40x). These features were also found on the lymph node (not shown); 6: the neoplasm consisted of irregular nodules of follicular dendritic cells (CD21, 40x); 7: extensive background of CD4+ T-lymphocytes (CD4, 40x); 8: intermingled scattered large “popcorn cells” could be observed (HE, 200x); 9: the neoplastic popcorn cells were positive for CD20 (CD20, 200x); 10: they were negative for CD30 (CD30, 200x) and CD15 (not shown).

The patient was a 76-year-old female followed up in the Thyroid Clinic for a thyroid nodule and primary hypothyroidism. The patient also had type 2 diabetes mellitus, arterial hypertension, and hypercholesterolemia. Regular medication included levothyroxine, ramipril, and simvastatin, diabetes being controlled with dietary measures only. Family and personal history were noncontributory and there was no previous radiation or iodine exposure. A thyroid nodule was first diagnosed upon clinical suspicion and ultrasound confirmation 2 years previous to her first Endocrinology Clinic visit. Imaging documented a large (29 × 44 × 31 mm transverse, longitudinal, and anteroposterior diameters, resp.), hypoechogenic, heterogeneous thyroid nodule of the left lobe with regular borders (Figure 1) superimposed on a micronodular background and multiple locoregional (left posterior and internal jugular) lymphadenopathies of large dimensions (the largest with 48 × 20 mm diameter) (Figure 2).

Laboratory studies were compatible with compensated primary hypothyroidism (TSH 2.32 IU/mL (normal range 0.4–4.68 IU/mL); free thyroxine 13 pmol/L (normal range 10–28 pmol/L)) along with marginally elevated thyroid autoantibody titer (antithyroid peroxidase, 71.8 IU/mL; negative antithyroglobulin results). No changes were seen on the complete blood count, lactate dehydrogenase, or erythrocyte sedimentation rate. No thyroid scintigraphy was done due to the absence of hyperthyroidism.

Fine needle aspirations (FNA) of the thyroid nodule were inconclusive; however, upon repetition, suspicion of papillary thyroid carcinoma (follicular and Hürthle cells with moderate degree anisokaryosis and nuclear grooves, Thy Class 4) on a thyroiditis background (mixed lymphoid population with predominance of small lymphocytes) arose. FNA of a suspicious lymph node was, on the other hand, suggestive of a lymphoproliferative disorder (monomorphic population of small lymphocytes and rare macrophages). Flow cytometry of both the thyroid nodule and a suspicious lymph node cytology specimen did not show any monoclonal lymphoid cells although there was a greatly increased CD4/CD8 ratio.

Upon suspicion of a differentiated thyroid cancer, total thyroidectomy with cervical lymph node dissection was performed.

- Intraoperative pathology consultation was requested for the thyroid specimen that showed a well delimitated, nonencapsulated, homogeneous, elastic, whitish nodule occupying the left thyroid lobe with 44 mm of greatest diameter. Frozen sections of the nodule revealed a nodular lymphoid proliferation suggestive of a malignant lymphoma. No evidence of papillary carcinoma was present. On paraffin sections the findings were similar to the those found on the lymph node.
- Regional cervical lymph node dissection revealed extensive HL involvement (16 out of 18 lymph nodes with evidence of HL). The lymph nodes had a macroscopically homogeneous whitish surface. Microscopy revealed architectural distortion induced by proliferation of multiple small nodules with irregular borders composed of dendritic cells (CD21+), numerous small CD4+ T lymphocytes, and few histiocytes and B lymphocytes. In this highly cellular background, a few large neoplastic cells with scant cytoplasm and large lobulated vacuolated nuclei with one or more red nucleoli (“popcorn cells”) were present. Very few mitotic figures could be seen and no necrosis could be appreciated. Immunohistochemistry of the neoplastic cells showed positivity for CD20 and negativity for CD30 and CD15 (Figure 2).* In situ* hybridization for EBV was negative.
- Diagnostic conclusion: both specimens were compatible with Nodular Lymphocyte Predominant Hodgkin's Lymphoma (NLPHL).

Bone marrow biopsy and full body CT-scan revealed no further evidence of extranodal disease. Taken together, these results allowed classifying our patient's HL in the stage IA.

Besides thyroidectomy and cervical lymphadenectomy, the patient subsequently underwent systemic chemotherapy with ABVD (doxorubicin, bleomycin, vinblastine, and dacarbazine). With treatment, a favorable neoplastic response was observed. A total of 5 cycles were completed. Unfortunately, because of significant cardiac toxicity from anthracyclines, hospital admission for acute congestive heart failure and significant performance status deterioration, chemotherapy was suspended (after 5 cycles). The patient is presently well and displays no evidence of disease recurrence, upon 30 months of follow-up.

## 3. Discussion

Thyroid malignancies are increasingly being diagnosed in the current era of the “*Incidentalomas*,” differentiated thyroid cancer accounting for the majority of them. Thyroid lymphomas are very rare [5–7], both primary and secondary forms. Primary Malignant Thyroid Lymphoma (TL) is a rare thyroid neoplasm representing less than 5% of all thyroid cancers and 2.5% of all NHL [10]. The peak incidence is in the 7th decade and affects more frequently the female gender. TL are almost always of B cell lineage. Most being initially MALT lymphomas, the majority may ultimately progress to Diffuse Large B Cell Lymphoma (DLBCL), which account for 70–80% of all TL at the time of diagnosis.

Concerning Primary Thyroid Lymphomas, Hashimoto thyroiditis is considered a risk factor predisposing to its development and, in fact, in up to 40% of cases, concomitant primary hypothyroidism has been reported. Clinically, compressive symptoms are present in one-third and accompanying locoregional lymphadenopathy in 50% of cases. Regarding therapy, surgery may be indicated in some cases despite chemotherapy (with or without Radiation therapy) the being most frequently employed treatment modality [9].

Our literature search revealed thirty-nine cases of Hodgkin's lymphoma located in the thyroid gland reported to date. Thyroid Hodgkin lymphoma affects mostly female patients (75–80% patients) with a median age of 42 years at the time of diagnosis [8]. Thyroid gland, a lymphoid cell deprived organ, requires an inflammatory insult for local lymphocyte allocation [11]. This is one theory supporting a possible association between lymphocytic thyroiditis and HL. In 80% of patients, thyroid HL became manifest as a rapidly enlarging neck mass, compressive symptoms being somewhat frequent, hoarseness (35%), dyspnea (65%), and dysphagia (53%). Classical B symptoms, fever (>38°C) for 3 or more days, drenching night sweats, and significant weight loss (>10% body weight in 6 months), are rare and may herald systemic involvement and disease progression, representing a predictor of poor outcome [3]. Classical Hodgkin's Lymphoma, namely, nodular sclerosis subtype, is the most frequently described variant to affect the thyroid gland [8].

Nodular lymphocyte predominant HL (NLPHL) constitutes a rare subtype of HL, accounting for no more than 10% of all cases of HL. It is considered a distinct clinical entity in that it differs from classical forms of HL in many aspects: Median age at presentation tends to be higher than in classical HL (30–40 years) and it has a greater male predominance (3 : 1). 70–80% of patients present with stage I-II disease and tend to have a higher propensity for peripheral lymph node involvement (neck and axilla). “B symptoms” are less frequent, being present in up to 10% of cases, which possibly reflects a lower systemic cytokine release (IL-2, -4, and -13). Extranodal involvement is rare, the spleen being the most commonly involved organ (10–15%). Bone marrow infiltration is found in less than 5% of cases.

Its hallmark histologic feature is the presence of malignant LP cells (histiocytic Reed-Sternberg cell variants) embedded within a nodular pattern of infiltrating lymphocytes. Malignant cells are described as “*popcorn cells*,” an illustrative description of their morphology (containing a single, large, folded, or multilobated nucleus with multiple small basophilic nucleoli). In contrast with Reed-Sternberg cells, LP cells do not express CD 15 or CD30, being rather positive for CD19, CD20, CD22, CD45, and CD79a along with expression of Oct-2 and B cell Oct-binding protein 1 transcription factors. Reed-Sternberg cells are identified in more than half of NLPHL cases; however, these tend to be scarce, positive for CD20, and negative for CD15 and CD30. Markers for EBV are typically negative.

Recommended management involves field or regional radiotherapy for patients with limited stage disease and combined modality therapy or chemotherapy for advanced stage disease. Response rates are very high, complete remission being achieved in 90–100% of patients. Relapses are unfortunately common within 3–6 years (10–35%); however, prognosis remains overall favorable (10-year survival rates of 80–90%) [12].

The patient described is, to our knowledge, the first documented case of nodular lymphocyte predominant HL involving the thyroid. Patient was older than was expected from what has been described with this HL subtype. Lymphocytic thyroiditis was definitely present which may have been a decisive risk factor and a possible factor underlying difficulty in establishing the definite diagnosis. Primary Malignant Lymphoma of the thyroid diagnosis was assumed postoperatively justifying the adjuvant postoperative conventional chemotherapy for HL (ABVD regimen). Lymphoma free survival is reported after 2 years of follow-up despite possible significant treatment toxicity (heart failure).

## 4. Conclusions

Thyroid nodules are frequent clinical and radiological findings, the majority being benign in nature. Among thyroid malignancies, lymphomas are quite rare and are usually of non-Hodgkin's subtype. Hodgkin lymphomas of the thyroid are even more unusual, only 40 cases being described so far (mostly of the nodular sclerosis subtype). Nodular lymphocyte predominant HL is considered to represent a distinct form of HL regarding epidemiological distribution, biological behavior, histological hallmarks, and prognosis. The authors report on the first described case of a nodular lymphocyte predominant Hodgkin lymphoma of the thyroid.

## Additional Points

*Take-Home Messages*. (i) Hodgkin lymphomas of the thyroid gland are very rare. (ii) Nodular sclerosis HL is the most frequent HL subtype described to affect the thyroid. (iii) Nodular lymphocyte predominant HL is a distinct form of HL and so far had not been reported to affect the thyroid. (iv) Thyroid HL usually presents as a space occupying lesion, B symptoms and systemic involvement being rare. (v) In this subtype of HL, Reed-Sternberg cells are rare and are immunophenotypically distinct (CD20 positive, CD15 and CD30 negative). (vi) Treatment options range from field or regional radiotherapy for limited stage disease to combined modality therapy or chemotherapy for advanced stage disease.
